# Moderating effect of alexithymia between problem gambling and psychotic experiences in university students

**DOI:** 10.1186/s12888-023-05472-7

**Published:** 2024-01-03

**Authors:** Feten Fekih-Romdhane, Farah Ghrissi, Manel Stambouli, Abir Hakiri, Alexandre Andrade Loch, Majda Cheour, Souheil Hallit

**Affiliations:** 1 The Tunisian Center of Early Intervention in Psychosis, Department of Psychiatry “Ibn Omrane”, Razi hospital, 2010 Manouba, Tunisia; 2 Faculty of Medicine of Tunis, Tunis El Manar University, Tunis, Tunisia; 3 Laboratorio de Neurociencias (LIM 27), Instituto de Psiquiatria, Hospital das Clinicas HCFMUSP, Faculdade de Medicina, Universidade de Sao Paulo, Sao Paulo, SP, BR Brazil; 4 Instituto Nacional de Biomarcadores em Neuropsiquiatria (INBION), Conselho Nacional de Desenvolvimento Cientifico e Tecnológico, Sao Paulo, Brazil; 5 School of Medicine and Medical Sciences, Holy Spirit University of Kaslik, Jounieh, P.O. Box 446, Lebanon; 6 Psychology Department, College of Humanities, Effat University, 21478 Jeddah, Saudi Arabia; 7 Applied Science Research Center, Applied Science Private University, Amman, Jordan

**Keywords:** Gambling problem, Psychotic experiences, Alexithymia, Students, Moderation

## Abstract

**Background:**

Most of the young individuals with problem gambling (PG) or psychotic experiences (PEs) are less prone to seek medical help. Therefore, community-based studies investigating the relationship between these entities in non-clinical young people across a continuum of severity are warranted. To this end, the present study proposes to advance knowledge on the mechanisms that potentially underlie the association between PG and PEs, by examining the role of a potential moderator, i.e. alexithymia, in this relationship.

**Methods:**

A total of 399 participants enrolled in this study (mean age = 21.58 ± 3.20 years) participated in an online cross-sectional survey. The South Oaks Gambling Screen (SOGS), the Prodromal Questionnaire-Brief (PQ-B), and the Toronto alexithymia scale (TAS-20) were used.

**Results:**

Thirty-three (8.3%) participants had problem-gambling, whereas 13 (3.3%) were probable pathological gamblers. Moderation analysis results adjusted over confounders (age, household crowding index, marital status, personal history of mental disorder, other illegal drug use) showed that the interaction PG by alexithymia (*p* = .018) was significantly associated with PEs scores. At moderate (Beta = 1.93) and high (Beta = 3.38) levels of alexithymia, more PG was significantly associated with more PEs scores.

**Conclusion:**

Findings suggest that GP may have a different impact on PEs depending on the individual’s level of alexithymia. As such, both alexithymia and gambling behavior should be considered in the clinical assessment of young people who present with PEs, which can help in implementing more tailored and individualized treatment plans.

## Introduction

As defined by the DSM-5, gambling disorder is characterized by persistent and recurrent gambling behavior despite adverse problems and distress [1]. It has a prevalence ranging from 0.7 to 6.5% during lifetime in different countries across the world [2]. A less severe, subclinical form of gambling disorder is referred to as problem gambling (PG) [3]. PG has been linked to elevated rates of multiple comorbid mental disorders [4–7], with a prevalence of up to 19% for schizophrenia and related psychoses [8–10]. Prior research has also outlined that compared to non-clinical individuals, those with psychosis are 3 to 4 times more likely to have PG [10]. At the same time, some epidemiological studies pointed to an increased risk of experiencing psychosis among problematic and impaired gamblers relative to the general population [11, 12]. Empirical studies showed that around 5% of high-risk gamblers meet the criteria for a psychotic disorder, which is much higher than prevalence rates observed in the general population [11, 13–17]. Based on these data, it appears that PG and psychosis tend to co-occur and may exacerbate each other [18]. However, until recently there were no studies available on the association between PG and milder forms of psychosis, including psychotic experiences (PEs). This has been considered as an important gap in the current literature, given the growing evidence connecting PG to the severe end of the psychosis spectrum [19].

PEs refer to symptoms of psychotic disorders conditions (i.e., hallucinations and delusions) with less intensity and persistence, which can be associated with distress and need for treatment [20], and can be commonly seen in the general population [21]. It was recently found in a systematic review that PEs affect around 7% of the general population, a prevalence around 10 times higher than that of schizophrenia (Saha et al., 2005). Individuals with PEs could be at risk for developing more severe forms of psychosis such as schizophrenia [22], as well as other physical [23] and mental [24] health problems. The first study on PEs and gambling was published in 2018, and based on cross-sectional data obtained from a large sample of UK adults from the general population [19]. This study found that the prevalence of PEs increased with gambling severity, with at-risk gambling being associated with a 2-fold and problem gambling with a 5-fold increase in the odds for PEs. The co-existence of gambling problems with PEs was not largely explained by substance use or common mental disorders [19]. As the way and extent to which individuals engaging in gambling could exhibit PEs and vice-versa is a relatively novel and expanding field, the mechanisms and pathways linking gambling and psychosis are unknown and remain to be elucidated. Investigating these mechanisms is highly relevant, especially since the association between gambling and psychosis was found to be related to elevated risk for other mental disorders comorbidities (e.g., addictive behaviors) [25], more severe PG, and worse psychopathology [14].

Multiple mechanisms can be proposed to explain the association between PG and PEs. One possible explanation can be the role of impulsivity, as it has proven to correlate with both conditions [26–29]. Another explaining factor that was also found to contribute to PG and PEs is psychological distress [30, 31]. Furthermore, it was found that gamblers are more likely to engage in maladaptive coping strategies [32], and that the latter may act as a mediator between stress and PEs [33], thus suggesting that maladaptive coping may be implicated in the association between gambling behavior and PEs. Cognitive distortions could be another underlying factor involved in the association between PG and PEs [34, 35]. In addition, there is some evidence that emotional dysregulations are more prevalent in gamblers compared to nongamblers [36], and may play a major role in the development of PEs [37]. In this regard, the present study proposes to advance knowledge on the mechanisms that potentially underlie the association between PG and PEs, by examining the role of a potential moderator, i.e. alexithymia, in this relationship.

### Alexithymia as moderator between PG and PEs

In this work, we made the theoretically-driven hypothesis that alexithymia may moderate the association between PG and PEs, based on the available evidence that high prevalence rate of alexithymia was found in both conditions [38, 39]. Alexithymia can be regarded as a trans-diagnostic personality dimension underscoring disorders of affect regulation [40]. It is characterized by a reduced ability of identifying, describing, analyzing, and differentiating one’s own and others’ inner emotional states [41, 42]. The development of alexithymia traits remains poorly understood, and has been observed by several theoretical perspectives, including psychoanalysis [43], attachment theory [44], and self-determination theory [45]. Alexithymia was regarded as a psychoanalytic construct consisting of a defense against neurotic conflicts and anxiety, rather than a type of affect deficit [43]. The attachment theory proposes that alexithymia can emerge as a result of dysfunctional attachment, then lead in turn to psychopathology [44]. The self-determination theory stipulates that alexithymia is related to needs fulfillment, suggesting that it may stem from both perceived controlling parenting (which represents a need-thwarting context) and the frustration of basic psychological needs [45].

On the one hand, previous studies found that individuals with gambling problems exhibit higher levels of alexithymia compared to healthy controls (e.g., [46–51]). A recent systematic review encompassing 20 papers (all conducted in Western countries) revealed that alexithymia is associated in a dose-response fashion with gambling-related problems in both community and clinical samples, with prevalence estimates of 31–52% in non-clinical pathological gamblers and 34–67% in people diagnosed with gambling disorder [38]. Accordingly, it has been suggested that gambling would emerge as an attempt of alexithymic individuals to self-regulate their emotions, namely to avoid negative emotions and increase emotional arousal [38].

On the other hand, alexithymia has been hypothesized to play a key role in the vulnerability to psychosis. Indeed, there is consistent evidence to support that individuals diagnosed with schizophrenia have heightened levels of alexithymia [52]. Additionally, alexithymia was suggested to be associated with psychotic symptoms in patients with schizophrenia [53, 54]. This relationship was shown to extend beyond people with the psychiatric disorder to people with attenuated or subthreshold forms of psychosis. For example, a study by van der Velde et al. [55] demonstrated that patients with schizophrenia and their siblings displayed higher levels of alexithymia compared to healthy controls, and that individuals at ultra-high risk (UHR) for psychosis had even higher levels of alexithymia than siblings, suggesting that “alexithymia varies parametrically with the degree of risk for psychosis”. Likewise, a Dutch study reported that UHR adolescents with higher levels of schizophrenia spectrum pathology appear to have more pronounced alexithymia [56]. Another study indicated that male siblings of schizophrenia patients, who are at genetic risk for developing the disease, had significantly more difficulties with verbalizing emotions than controls [53]. More recently, alexithymia was shown to be linked to psychotic manifestations among adolescents [57].

### The present study

Due to its growing accessibility and availability, as well as its increasing prevalence [58, 59], gambling has become one of the most frequent addictive behaviors during adolescence and young adulthood [29]. For instance, a systematic review found prevalence rates of 0.2–12.3% of problem gambling among adolescents and young people (aged 10 to 24 years) [60]. This age group is the peak time of onset for both risky gambling behavior [61, 62] and psychotic disorders [63]. However, most of the young individuals with PG or PEs are less prone to seek medical help [64, 65]. Therefore, community-based studies investigating the relationship between these entities in non-clinical young people across a continuum of severity are warranted. To this end, this study sought to explore the interplay between gambling, PEs, and alexithymia after adjusting over socioeconomic status, as this factor was proven to be closely associated with PEs [66–68]. In particular, it aimed to test the hypothesis that Alexithymia will play a moderating role in the association between gambling and PEs such that an increase in Alexithymia will make this association stronger.

## Methods

### Sample and procedure

Eligible participants were: (1) university students originating from, and residing in Tunisia, (2) aged 18–35 years (as the at-risk for psychosis population predominantly belongs to this age range [69]), and (3) who consented to participate. Participants were invited to take part in an online survey during the period from January to April 2023. All participants were asked to respond to a self-administered, anonymous questionnaire created on Google forms in the Arabic language. Thereafter, the link was shared to other potential participants through social networks using the snowball technique. To reach the largest possible sample size, the link to the questionnaire was sent to friends and family members of the research team, who were asked in turn to forward the link to their family members, friends and their contact list via social media networks (including Facebook, Instagram, and WhatsApp). The questionnaire took around 20 min to be completed. All participants were informed of the procedures and objectives of the research and that their consent was voluntary. To ensure anonymity and confidentiality, respondents’ names were not requested. A total of 399 valid responses were received. The protocol was approved by the ethics committee of Razi Hospital, Manouba, Tunisia, before the start of the study. The study was conducted following the Declaration of Helsinki for human research.

### Minimum sample size required

The G-power software v.3.0.10 (multiple regression option) estimated a minimum sample of 341 participants needed to have enough statistical power for the moderation analysis, based on a R^2^ deviation of 5% from zero, an alpha error of 5%, a power of 80% and 12 predictors to be entered in the multivariable model.

### Measures

Data were collected using a self-report, web-based questionnaire involving two sections. The first section contained questions covering sociodemographic information. Participants were asked about their age, gender, marital status (Married, Single, Divorced, Separated, Widowed), living area (Urban, Rural), substance use (Yes, No), personal history of mental illness (Yes, No), household crowding index (HCI), and self-perceived financial satisfaction. In addition, household crowding index (HCI), which reflects the socioeconomic status (SES) of participants, was calculated by dividing the number of persons by the number of rooms in the house [70]. Higher HCI indicate lower SES. The second section contained three measures: The South Oaks Gambling Screen (SOGS), the Prodromal Questionnaire-Brief (PQ-B), the Toronto alexithymia scale (TAS-20).

#### The SOGS

This measure contains 20 items (e.g., “When you gamble, how often do you go back another day to win back money you have lost?”) and represents the most widely used tool to assess pathological gambling. The scale was developed by Lesieur and Blume in 1987 [71]. Its format enables many ways of administration including self-report. A score of 1 to 4 indicates some problem with gambling, while a score of 5 or more indicates probable pathological gambling. The Arabic validated version of the SOGS demonstrated good psychometric properties [72]. Cronbach’s alpha in this study was of 0.904.

#### The PQ-B

This is a 21-item measure used to assess attenuated psychotic symptoms [73, 74]. Sample items are “Do familiar surroundings sometimes seem strange, confusing, threatening, or unreal to you?” and “Have you heard unusual sounds like banging, clicking, hissing, clapping, or ringing in your ears?”. All items are rated as yes or no; those who answered yes were asked to rate the level of distress caused by each statement on a 5-point scale ranging from 1 (strongly disagree) to 5 (strongly agree). We used the Arabic validated version of the PQ-B [75]. In the present sample, alpha Cronbach coefficient was of 0.923.

#### The TAS-20

The Arabic version of the TAS-20 was used [76]. It is composed of 20 items (e.g., “I am often confused about what emotion I am feeling” or “It is difficult for me to find the right words for my feelings”) assessing levels of alexithymia. Each item is scored on a 5-point Likert-type scale ranging from 1 (strongly disagree) to 5 (strongly agree). The TAS-20 demonstrated acceptable reliability and validity [77]. Alpha Cronbach was of 0.840.

### Statistical analysis

The SPSS software v.26 was used for the statistical analysis. The PQ-B score was considered normally distributed since the skewness and kurtosis values varied between ± 1.96. The Student *t* test was used to compare two means and the Pearson test to correlate two continuous variables. The moderation analysis was conducted using PROCESS MACRO (an SPSS add-on) v3.4 model 1 [78], taking alexithymia as a moderator in the association between gambling and PQ-B. Results were adjusted over all variables that showed a *p* < .25 in the bivariate analysis. We considered the mediation analysis to be significant if the Boot Confidence Interval did not pass by zero. *P* < .05 was deemed statistically significant.

## Results

A total of 399 participants enrolled in this study (mean age = 21.58 ± 3.20 years). Thirty-three (8.3%) participants had problem-gambling, whereas 13 (3.3%) were probable pathological gamblers. Other characteristics of the sample can be found in Table 1.

**Table 1:** Sociodemographic and other characteristics of the participants (n = 399)

Variable	n (%)
**Gender**	
Male	299 (74.9%)
Female	100 (25.1%)
**Marital status**	
Single / divorced	294 (94.3%)
Married	18 (5.8%)
**Living area**	
Urban	365 (91.5%)
Rural	34 (8.5%)
**Smoking**	
No	332 (83.2%)
Yes	67 (16.8%)
**Alcohol drinking**	
No	325 (81.5%)
Yes	74 (18.5%)
**Cannabis use**	
No	371 (93.0%)
Yes	28 (7.0%)
**Other illegal drug use**	
No	386 (96.7%)
Yes	13 (3.3%)
**Personal history of mental illness**	
No	226 (56.6%)
Yes	173 (43.4%)
	**Mean ± SD**
Age (years)	23.80 ± 5.00
Household crowding index	1.26 ± 0.66
Financial satisfaction	5.00 ± 2.83
PQB score	24.68 ± 20.94
Alexithymia total score	59.45 ± 12.93
SOGS total score	0.40 ± 1.44

### Bivariate analysis

The bivariate analysis results are shown in Tables 2 and 3. A higher PQ-B score was significantly found in participants who were single compared to married, in those who used other illegal drugs than cannabis and had a personal history of mental illness compared to not. Moreover, older age was significantly associated with lower PQ-B scores, whereas a higher household crowding index, alexithymia and SOGS scores were significantly associated with higher PQ-B scores.

**Table 2:** Bivariate analysis of the categorical variables and PQB scores

Variable	Mean ± SD	*t*	*df*	*p*
**Gender**		− 0.210	397	0.834
Male	24.55 ± 20.59			
Female	25.06 ± 22.05			
**Marital status**		3.08	397	**0.002**
Single / divorced	25.37 ± 21.10			
Married	10.00 ± 8.28			
**Living area**		1.114	397	0.266
Urban	25.04 ± 21.01			
Rural	20.85 ± 20.00			
**Smoking**		− 0.879	397	0.380
No	24.27 ± 20.32			
Yes	26.73 ± 23.81			
**Alcohol drinking**		0.137	397	0.891
No	24.75 ± 21.02			
Yes	24.38 ± 20.69			
**Cannabis use**		-1.030	397	0.304
No	24.38 ± 20.76			
Yes	28.61 ± 23.22			
**Other illegal drug use**		-2.981	397	**0.003**
No	24.11 ± 20.27			
Yes	41.54 ± 32.25			
**Personal history of mental illness**		-7.813	397	**< 0.001**
No	18.00 ± 17.34			
Yes	33.40 ± 22.04			

Numbers in bold indicate significant *p* values

**Table 3:** Correlation matrix of continuous variables

Variable	1	2	3	4	5	6
1. PQB	1					
2. Age	− 0.17**	1				
3. Household crowding index	0.14**	− 0.08	1			
4. Financial satisfaction	− 0.03	0.04	− 0.06	1		
5. Alexithymia	0.50***	− 0.22***	0.02	− 0.06	1	
6. SOGS	0.24***	0.05	− 0.05	0.002	0.16**	1

Numbers in the table reflect Pearson correlation coefficients; **p < .01; ***p < .001

### Moderation analysis with psychological distress taken as the dependent variable

**Fig. 1:**
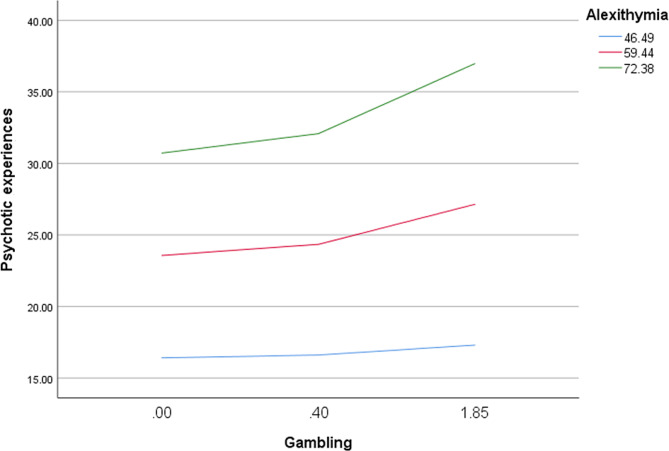
Interaction between problem gambling and alexithymia on psychotic experiences

The details of the moderation analysis of alexithymia taken as a moderator in the association between PG and PQ-B, are summarized in Table 4. The results were adjusted over age, household crowding index, marital status, personal history of mental disorder, other illegal drug use. The interaction PG by alexithymia (*p* = .018) was significantly associated with PQ-B scores (Table 4); at moderate (Beta = 1.93) and high (Beta = 3.38) levels of alexithymia, more PG was significantly associated with more PEs (Table 5; Fig. 1).

**Table 4:** Moderation analysis taking problem gambling as the independent variable, alexithymia as the moderator and psychotic experiences as the dependent variable

Moderator	Beta	*t*	*P*	95% CI
PG	-4.72	-1.49	0.138	-10.97; 1.52
Alexithymia	0.55	7.52	**< 0.001**	0.41; 0.70
Interaction PG by alexithymia	0.11	2.37	**0.018**	0.02; 0.21*

PG: Problem Gambling

*indicates significant moderation; numbers in bold indicate significant *p* values; results adjusted over age, household crowding index, marital status, personal history of mental disorder, other illegal drug use

**Table 5:** Conditional effects of the focal predictor (problem gambling) at values of the moderator (alexithymia)

Alexithymia	Beta	*t*	*p*	95% CI
Low (= 46.49)	0.48	0.44	0.661	-1.68; 2.65
Moderate (= 59.44)	1.93	2.86	**0.005**	0.60; 3.26
High (= 72.38)	3.38	5.05	**< 0.001**	2.07; 4.70

Numbers in bold indicate significant *p* values

## Discussion

Studies drawing reliable empirical evidence on the specific mechanisms, in particular individual vulnerability factors, linking PG to PEs in community young adults are important from a public health perspective, and may deepen our knowledge of the developmental pathways to psychosis. This study aimed to contribute to the understanding of the association between PG and PEs, by exploring the moderating role of alexithymia. Findings showed that alexithymia partially moderated the effect that gambling exerted on PEs. This effect emerged for students who reported moderate to high levels of alexithymia. It could, therefore, be speculated that GP may have a different impact on PEs depending on the individual’s level of alexithymia.

The first expected finding was that PG was significantly and positively associated with the severity of PEs among our students. The majority of previous literature on this research topic has emerged from the Western world, and was mainly focused on clinical populations and the extreme end of the severity continuum of both conditions (i.e., gambling disorders and schizophrenia [9, 11–13, 16, 17]). It is only recently that the association between the milder forms of the diseases has been investigated and established in UK community adults [19]. Findings revealed that PG was related to a 5-fold higher risk of reporting PEs [19]. The co-occurrence of gambling with psychosis was shown to exacerbate the severity of PG, increase the likelihood of comorbidity with other mental disorders (including depression and anxiety disorders), as well as lifetime suicidality [11]. Therefore, future studies from different backgrounds are needed to further explore this relationship.

Moderating analyses showed that alexithymia strengthened the association between gambling and PEs. From a theoretical perspective, the moderating role of alexithymia was driven from the evidence that the alexithymic deficit in processing feelings is likely to contribute to both PG and PEs. Our finding is broadly consistent with previous studies in which highly alexithymic individuals reported worse mental health outcomes after engaging in gambling (e.g., [79]). The present finding is helpful for clinicians and mental health workers in further understanding the psychopathological factors underlying the relationship gambling-psychosis, and identifying groups that are more vulnerable to psychosis when gambling problems appear. It can be suggested that improving alexithymia in young individuals who encounter gambling problems may decrease PEs, which provides a novel perspective for prevention and early intervention in psychosis. More particularly, interventions having shown effectiveness in reducing and improving alexithymia (such as mindfulness-based interventions [80], or dialectical behavior therapy-based interventions [81]) might help clinicians better mitigate and prevent the onset or exacerbation of PEs among vulnerable gamblers.

### Clinical and research implications

The current results provided additional support to the positive association between PG and PEs among community young adults. In light of previous literature and the present findings, it appears of utmost importance to mitigate the possible negative effects of the dual presence of pathological gambling with PEs through evidence-based integrative intervention plans. One promising avenue suggested by our results may be alexithymia. Mediation results suggest that individuals with higher alexithymia who had gambling problems exhibited more severe PEs. Therefore, clinicians should be alert to the possibility of PEs when working with alexithymic youth having gambling problems. Furthermore, PG was shown to positively respond to multiple strategies of care [82], and alexithymia may also be effectively lowered with psychological interventions [83]. In particular, a previous systematic search of the literature has shown that specific psychotherapeutic techniques, such as body-centered psychotherapy, may help individuals with gambling problems and high alexithymia to develop more adaptive strategies to effectively regulate and manage their inner emotional states [84]. As such, both alexithymia and gambling behavior should be considered in the clinical assessment of young people who present with PEs, which can help in implementing more tailored and individualized treatment plans. In addition, our study opens the door to identifying other potential moderators implicated in the relationship between gambling and psychosis in future research, such as impulsivity or cognitive distortions.

### Study limitations

The present findings need to be interpreted while considering some limitations. First, data were gathered using online survey, which has mostly attracted females (74.9%) from urban areas (91.5%). This may likely limit the representativeness of our sample. Second, a cross-sectional design as adopted, which precludes the ability to draw causal inferences. Future longitudinal studies are required to confirm the present findings. Third, only university students were involved in this study, which may limit the generalization of our results to the broader population of community young adults. Fourth, self-report measures were used, therefore, findings may be prone to social desirability bias.

## Conclusion

To conclude, findings revealed a positive link between PG and PEs among Tunisian university students. Clinicians need to be aware of the possible co-occurrence of these two conditions in young adults. Given the potential detrimental implications of the association between gambling and psychosis, additional research of longitudinal design is needed to further understand the mechanisms underlying this relationship. In addition, future experimental studies are required to explore ways how to address psychotic symptoms in gamblers. In particular, these studies need to test the effectiveness of interventions targeting alexithymia in improving PEs among gamblers.

## Data Availability

The datasets generated and/or analyzed during the current study are not publicly available due to restrictions from the ethics committee but are available from the corresponding author on reasonable request.
